# Angiotensin II-induced endothelial dysfunction is temporally linked with increases in interleukin-6 and vascular macrophage accumulation

**DOI:** 10.3389/fphys.2014.00396

**Published:** 2014-10-29

**Authors:** Jessica R. Gomolak, Sean P. Didion

**Affiliations:** ^1^Department of Pharmacology, The University of Mississippi Medical CenterJackson, MS, USA; ^2^Department of Neurology, The University of Mississippi Medical CenterJackson, MS, USA

**Keywords:** carotid artery, endothelium, interleukin-6, vascular hypertrophy, mice

## Abstract

Angiotensin II (Ang II) is associated with vascular hypertrophy, endothelial dysfunction and activation of a number of inflammatory molecules, however the linear events involved in the development of hypertension and endothelial dysfunction produced in response to Ang II are not well defined. The goal of this study was to examine the dose- and temporal-dependent development of endothelial dysfunction in response to Ang II. Blood pressure and responses of carotid arteries were examined in control (C57Bl/6) mice and in mice infused with 50, 100, 200, 400, or 1000 ng/kg/min Ang II for either 14 or 28 Days. Infusion of Ang II was associated with graded and marked increases in systolic blood pressure and plasma Ang II concentrations. While low doses of Ang II (i.e., 50 and 100 ng/kg/min) had little to no effect on blood pressure or endothelial function, high doses of Ang II (e.g., 1000 ng/kg/min) were associated with large increases in arterial pressure and marked impairment of endothelial function. In contrast, intermediate doses of Ang II (200 and 400 ng/kg/min) while initially having no effect on systolic blood pressure were associated with significant increases in pressure over time. Despite increasing blood pressure, 200 ng/kg/min had no effect on endothelial function, whereas 400 ng/kg/min produced modest impairment on Day 14 and marked impairment of endothelial function on Day 28. The degree of endothelial dysfunction produced by 400 and 1000 ng/kg/min Ang II was reflective of parallel increases in plasma IL-6 levels and vascular macrophage content, suggesting that increases in arterial blood pressure precede the development of endothelial dysfunction. These findings are important as they demonstrate that along with increases in arterial pressure that increases in IL-6 and vascular macrophage accumulation correlate with the impairment of endothelial function produced by Ang II.

## Introduction

It is well documented that angiotensin II (Ang II), the main effector peptide of the renin-angiotensin system, produces hypertension, oxidative stress, vascular hypertrophy, and endothelial dysfunction (Bean et al., 1979; Brown et al., 1981; Didion et al., 2002, 2005; Didion and Faraci, 2003; Cassis et al., 2004; Crowley et al., 2005; Schrader et al., 2007). In humans, several forms of hypertension are associated with increases in plasma Ang II levels, such as renovascular hypertension and hypertension due to renin secreting tumors (Catt et al., 1969, 1971; Garovic and Textor, 2005; Beevers et al., 2008). Genetic and experimental models, such as mice which express human renin and angiotensinogen, renovascular hypertension (e.g., 2-kidney, 1-clip) and infusion of exogenous Ang II, are commonly used approaches to model human hypertension associated with increases in Ang II (Bean et al., 1979; Johns et al., 1996; Didion et al., 2000, 2002, 2005).

In the last couple of decades the use of Ang II infusion has increased dramatically. It has been estimated that infusion of Ang II is the most commonly used model of hypertension as evidenced by use in nearly half of all NIH grants involving the use of an experimental model of hypertension (Galis et al., 2013). The popularity of the Ang II infusion model has been driven in part, due to the ease of use, but also due to the advent of technologies associated with the generation of genetically-altered mice and more recently genetically-altered rats (Smithies and Kim, 1994; Geurts et al., 2009). The combination of the Ang II model and genetic technologies has provided a wealth of information regarding genes that contribute to the development of hypertension and related vascular sequalae, including the role of oxidative stress and inflammatory cytokines as well as inflammatory cell types in promoting vascular hypertrophy and endothelial dysfunction (Bush et al., 2000; Wang et al., 2001; Ryan et al., 2004; Didion et al., 2005, 2009; Guzik et al., 2007; Schrader et al., 2007; Madhur et al., 2010; Barhoumi et al., 2011).

Recently, it has been suggested that there may be an overreliance on the Ang II model considering that the model is not reflective of primary (essential) hypertension, which accounts for the majority of hypertension in humans (Galis et al., 2013). While the Ang II infusion model is most reflective of secondary forms of hypertension, it should be noted that a major target of antihypertensive therapy in essential hypertension in humans has been directed toward limiting Ang II signaling (e.g., ACE inhibitors and angiotensin receptor blockers) (Sica, 2010; Hanselin et al., 2011). Thus, the Ang II infusion model clearly has direct relevance to human hypertension irrespective of etiology.

As with any model of human disease, there are both strengths and limitations associated with the Ang II infusion model. For example, Ang II infusion can produce immediate effects on blood pressure, which is a strength of the model, but also a limitation, as hypertension in humans usually develops over decades rather than minutes or days. The use of high doses of Ang II, particularly in the mouse, has been questioned as it relates to plasma concentrations observed in human hypertension. While is difficult to extrapolate between studies in different species, any potential comparison is complicated by the fact that in the majority of studies with Ang II infusion in mice either do not measure or do not report plasma Ang II levels (Bush et al., 2000; Ryan et al., 2004; Lee et al., 2006; Guzik et al., 2007; Didion et al., 2009; Barhoumi et al., 2011). Another limitation is that it is often difficult to make direct comparisons between studies, as there is marked variability between studies in terms of the dose (anywhere from 490 up to as high as 3600 ng/kg/min Ang II) and the length of time (as short as 3, 7, or 10 days and as long as 28 days) in which Ang II is infused (Bean et al., 1979; Didion et al., 2005, 2009; Lee et al., 2006; Guzik et al., 2007; Schrader et al., 2007; Madhur et al., 2010; Barhoumi et al., 2011). This is also complicated by the fact that the majority of studies in the literature examine single doses at single time points thus providing limited insight into the temporal development of hypertension and vascular dysfunction produced in response to Ang II (Bush et al., 2000; Ryan et al., 2004; Lee et al., 2006; Guzik et al., 2007; Didion et al., 2009; Barhoumi et al., 2011).

We had several goals for the present study. First, we sought to determine the effects of Ang II on blood pressure and plasma Ang II levels. Second, we sought to determine whether hypertension *per se* promoted the impairment of endothelial function or vice versa. Finally, we sought to determine the sequential and temporal activation of inflammatory molecules in the development of endothelial dysfunction in response to Ang II. We focused our studies of vascular function to the carotid artery, as carotid artery disease and hypertension are both important risk factors for ischemic stroke (Go et al., 2013; Kernan et al., 2014).

## Methods

### Animals

Male C57Bl/6 mice (#000664; *n* = 46) were obtained from the Jackson Laboratory (Bar Harbor, ME) and were randomly selected to either one of two groups (i.e., control or Ang II-infused group). Mice selected to the Ang II-infused group were implanted with either a 14 Day (Alzet^®^ Model 1002; DURECT™ Corporation, Cupertino, CA) or a 28 Day (Alzet^®^ Model 1004) micro-osmotic pump containing 50, 100, 200, 400, or 1000 ng/kg/min Ang II as described previously (Didion et al., 2005; Schrader et al., 2007). All experimental protocols conform to the *NIH Guide for the Care and Use of Laboratory Animals* and were approved by the Institutional Animal Care and Use Committee at the University of Mississippi Medical Center.

### Systolic blood pressure measurements

Systolic blood pressure was measured using tail-cuff plethysmography (Visitech Systems BP-2000 *Series II Blood Pressure Analysis System*™, Apex, NC) in a manner similar to that described previously by our laboratory (Didion et al., 2005; Schrader et al., 2007). Briefly, mice were trained for a period of 5 consecutive days in which blood pressure was measured (30 measurements per day) but not recorded. Following this training period, blood pressure was measured for another 3 consecutive days, recorded and averaged. The blood pressure measured during this period is reported as baseline (Day 0). Blood pressure was then measured on Days 3, 7, 14, 21, and 28 in both control and Ang II-infused groups.

### Studies of endothelial function in carotid artery

Depending on whether mice were implanted with a 14 or 28 Day minipump they and their respective controls were euthanized on either Day 14 or 28 with pentobarbital sodium (150 mg/kg ip). Arterial blood was collected for measurements of plasma Ang II and IL-6 levels. Bestatin^®^ (Sigma Chemical, St Louis, MO) was added to the blood samples in order prevent proteolysis of angiotensinogens. Both common carotid arteries as well as the thoracic aorta were carefully dissected and removed for studies of endothelial function as well as measurement of vascular macrophage content and medial cross-sectional area (CSA), respectively.

For studies of endothelial function, carotid arteries were placed in Kreb's buffer with the following composition (mmol/L): NaCl 118.3, KCl 4.7, CaCl_2_ 2.5, MgSO_4_ 1.2, KH_2_PO_4_ 1.2, NaHCO_3_ 25, glucose 11. Loose connective tissue was removed and then each carotid artery was cut into two vascular rings each 3–4 mm in length. Vascular rings were connected to a force transducer to measure isometric tension (contraction and relaxation) in individual organ baths containing 20 ml Krebs buffer maintained at 37°C and gassed with a mixture of 95% O_2_ and 5% CO_2_. Following a 45-min equilibration period vessels were pre-contracted (50–60% of maximum) with the thromboxane analog, 9,11-dideoxy-11a-epoxymethanprotaglandin F2α (U46619). After achieving a stable contraction plateau, concentration-response curves were generated to acetylcholine (an endothelium-dependent agonist; 0.01–100 μmol/L) and to nitroprusside (an endothelium-independent agonist; 0.01–100 μmol/L). Concentration-response curves were recorded using PowerLab^®^ data acquisition systems and analyzed off-line with LabChart^®^ software (ADInstruments, Colorado Springs, CO).

### Measurement of vascular hypertrophy

For measurements of medial CSA aortas were fixed in 4% buffered paraformaldhyde and then processed and embedded in paraffin while maintaining vessels in a cross-sectional orientation. Aortas were sectioned (7 microns), placed on microscope slides, stained with hematoxalin and eosin and then cover slipped. Sections were viewed and imaged using a Nikon Eclipse N*i*-U microscope. Medial CSA of the carotid artery was determined using Nikon NIS-Elements software as described previously (Schrader et al., 2007).

### Measurement of plasma Ang II and IL-6 levels

Plasma levels of Ang II and IL-6 were measured using a high-sensitivity mouse Ang II (Enzo, New York, NY) and IL-6 (R&D Biosciences, Minneapolis, MN) ELISA kit, respectively, according to manufacturer's instructions.

### Measurement of vascular macrophages

For measurement of vascular macrophage content, anterior segments of the left common carotid artery were frozen in optimal cutting temperature (O.C.T., Tissue-Tek, Sakura Finetek, USA) compound. Carotid arteries were sectioned using a cryostat and placed on glass microscope slides, boiled for 20 min in 10 mM citrate buffer, and blocked in 5% goat serum for 1 h at room temperature. Slides were incubated with a general macrophage (CD68) primary antibody (Abcam, ab955) overnight at 4°C. Prior to the horseradish peroxidase conjugated secondary antibody incubation slides were treated with 0.3% hydrogen peroxide followed by incubation with 3,3′-diaminobenzidine (DAB) for 10 min. Slides were counterstained with hematoxylin and CD68 positive cells were examined and counted (at a magnification of 40x) using light microscopy (Nikon Eclipse N*i* microscope, Tokyo, Japan).

### Drugs

Acetylcholine, Ang II (human, acetate salt), and nitroprusside were obtained from Sigma (St. Louis, MO) and were dissolved in saline. U46619 (in methyl acetate) was obtained from Cayman Chemical (Ann Arbor, MI) and evaporated with 100% nitrogen gas and re-suspended in 100% ethanol with subsequent dilutions being made with saline. All other reagents were of standard laboratory grade.

### Statistical analysis

All data are expressed as Means ± SE. Comparisons of blood pressure were made using Two-Way ANOVA. Responses to acetylcholine and nitroprusside are expressed as a percent relaxation to U46619-induced contraction and were compared using Two-Way ANOVA for repeated measures followed by Bonferroni *post-hoc* test. Medial cross-sectional area, plasma Ang II and IL-6 concentration, as well as vascular macrophage content were compared using One-Way ANOVA. Statistical significance was accepted at *P* < 0.05.

## Results

### Ang II infusion is associated with dose-dependent increases in blood pressure

**Figure 1:**
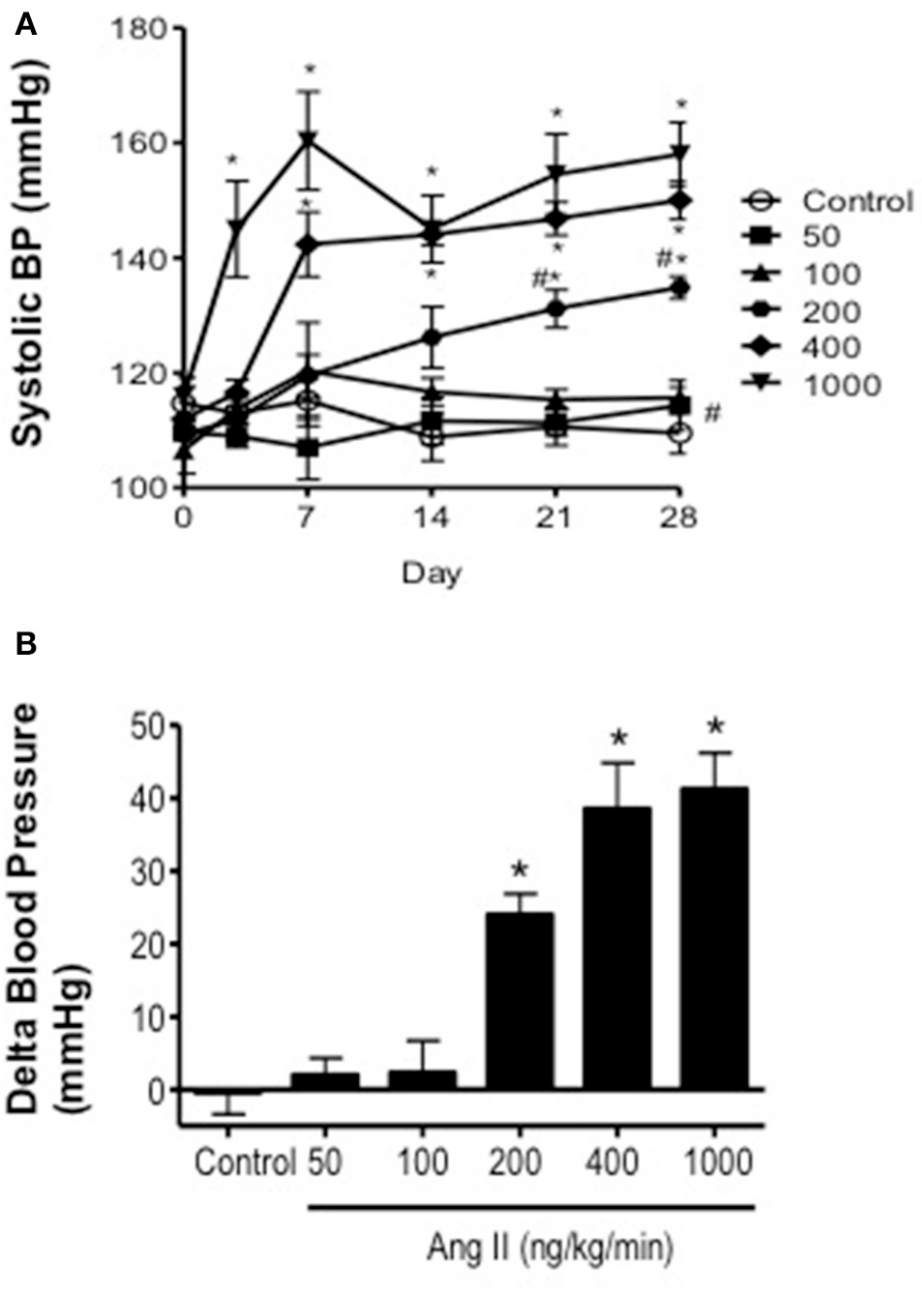
**Ang II infusion produces dose-dependent increases in systolic blood pressure. (A)** Systolic blood pressure over the course of 28 days in control and Ang II-infused mice as measured using tail-cuff plethysmography. **(B)** Delta change in blood pressure on Day 28 in response to graded doses of Ang II. Means ± SE; *n* = 3–8/group; ^*^P < 0.05 vs. Control; ^#^*P* < 0.05 vs. 1000 ng/kg/min.

Baseline systolic blood pressure was similar (*P* > 0.05) in all groups of mice prior to infusion of Ang II (Day 0) and collectively averaged 116 ± 5 mmHg. Ang II infusion was associated with a graded pressor response that was dependent on both the dose and length of time of Ang II infusion (Figure 1). For example, infusion of either 50 or 100 ng/kg/min Ang II had no effect (*P* > 0.05) on systolic blood pressure. Blood pressure in mice infused with 50 and 100 ng/kg/min was similar to that in control mice on Days 14 and 28. Infusion of 200 ng/kg/min Ang II was associated with a gradual increase in systolic pressure, which began by Day 14 and reached a maximum of 134 ± 2 mmHg on Day 28. Infusion of 400 ng/kg/min Ang II was also associated with a gradual increase in blood pressure that began on Day 7 reaching a maximum of 150 ± 3 mmHg on Day 28 (Figure 1). In contrast, Infusion of Ang II at a rate of 1000 ng/kg/min was associated with a marked and immediate increase in blood pressure (136 ± 6 mmHg on Day 3) that was maintained throughout the infusion period reaching a maximum of 158 ± 6 mmHg on Day 28.

### Ang II infusion is associated with dose-dependent increases in plasma Ang II levels

**Figure 2:**
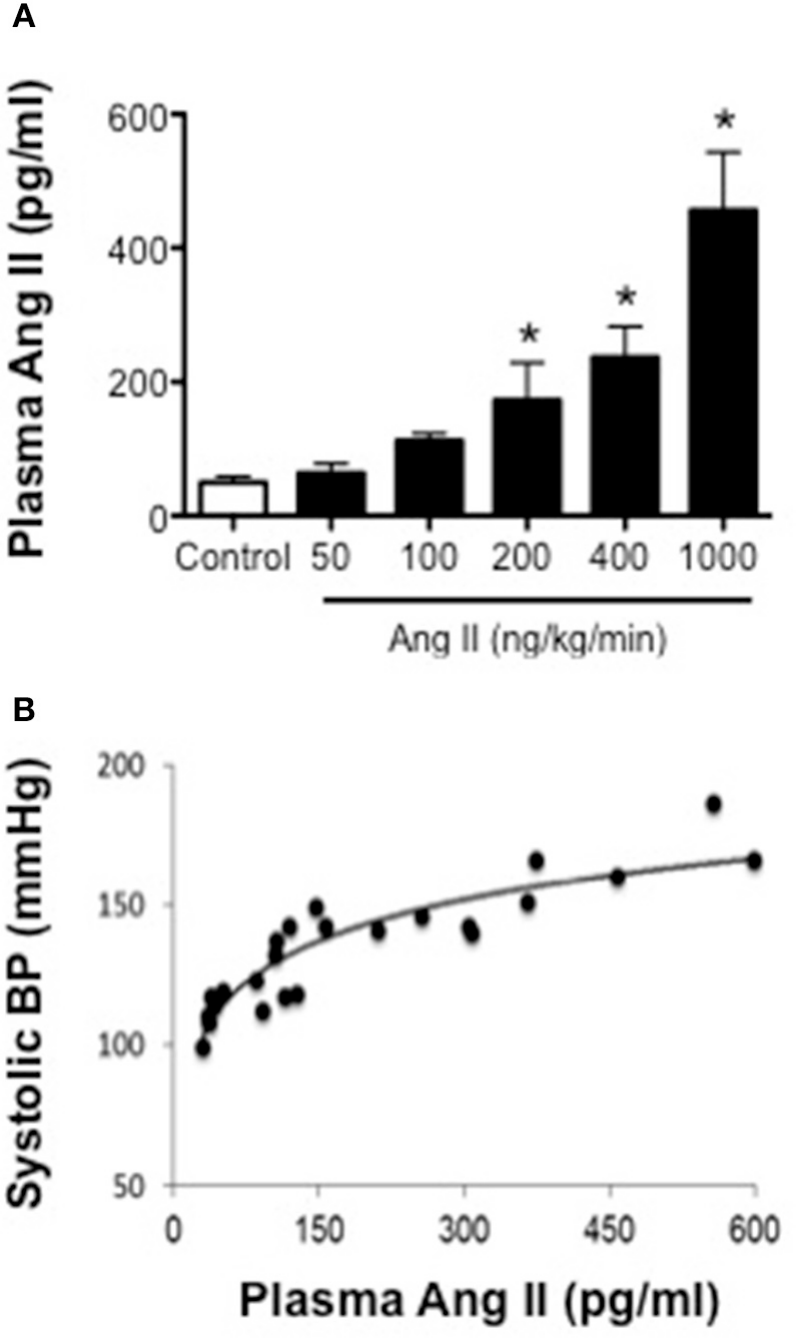
**Systolic blood pressure is positively associated with plasma Ang II levels. (A)** Plasma Ang II levels as measured by ELISA on Day 28. **(B)** Plasma Ang II concentration-blood pressure response curve (*R*^2^ = 0.8206). Means ± SE; *n* = 3–8/group; ^*^P < 0.05 vs. Control.

Ang II levels in control mice were 49 ± 12 pg/ml (Figure 2). In mice infused with 50 and 100 ng/kg/min Ang II, plasma Ang II levels were similar (*P* > 0.05) to that in control mice and averaged 63 ± 12 and 112 ± 7 pg/ml, respectively. Infusion of 200, 400, and 1000 ng/kg/min Ang II was associated with dose-dependent increases in plasma Ang II (Figure 2). For example, plasma Ang II levels in mice infused with 200 and 400 ng/kg/min were 173 ± 56 and 237 ± 46 pg/ml, respectively and were significantly different (*P* < 0.05) from Ang II levels in control mice but were not different (*P* > 0.05) from each other. Infusion of 1000 ng/kg/min Ang II was associated with the greatest increase in plasma Ang II levels (i.e., 456 ± 106 pg/ml), which was significantly higher than that in either control mice or any of the other Ang II-infused groups (Figure 2). When plasma Ang II levels were plotted against blood pressure, we found that there was a strong correlation between plasma Ang II levels and systolic blood pressure (Figure 2).

### Ang II infusion is associated with dose- and time-dependent reductions in endothelial function

**Figure 3:**
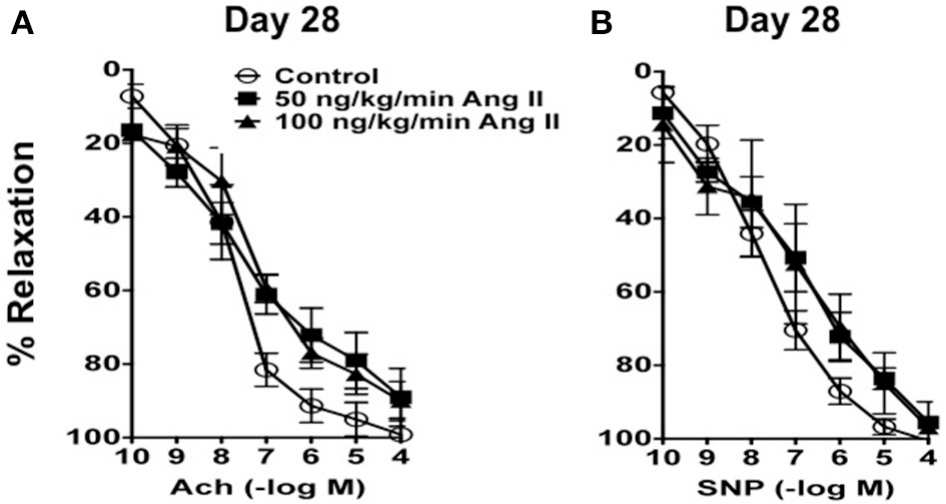
**(A)** Endothelium-dependent responses to acetylcholine (Ach) and **(B)** Endothelium–independent responses to nitroprusside (SNP) in carotid arteries from control mice and mice infused with 50 or 100 ng/kg/min of Ang II for 28 days. Means ± SE; *n* = 3/group; *P* > 0.05.

**Figure 4:**
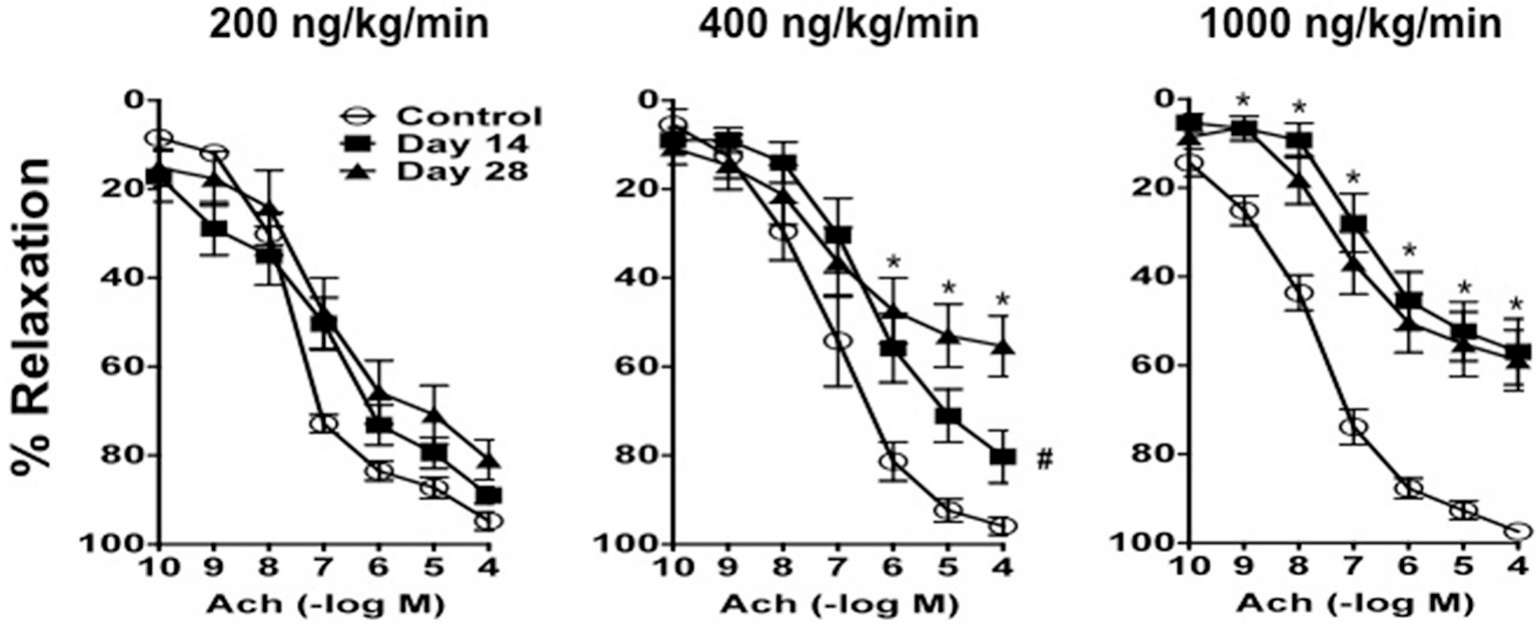
**Ang II produces impairment of endothelial function in a dose- and time-dependent manner**. Responses to acetylcholine (Ach) in carotid arteries from control mice and mice infused with 200 ng/kg/min, 400 ng/kg/min, or 1000 ng/kg/min of Ang II for 14 or 28 days. Means ± SE; *n* = 6–8/group; ^*^P < 0.05 vs. Control; ^#^*P* < 0.05 vs. Day 28.

In carotid arteries from control mice, acetylcholine produced concentration-dependent relaxation that was similar on Day 14 as that observed on Day 28 (Figures 3, 4). For example, 100 μmol/L acetylcholine produced 95 ± 3 and 97 ± 2% relaxation in carotid arteries from control mice on Day 14 and 28 respectively. Acetylcholine also produced relaxation in mice infused with 50 or 100 ng/kg/min of Ang II that was similar (*P* > 0.05) to that in control mice both on Day 28 (Figure 3). For example, 100 μmol/L acetylcholine produced 89 ± 7% relaxation in mice infused with 50 ng/kg/min Ang II and 100 ± 4% relaxation in mice infused with 100 ng/kg/min Ang II on Day 28.

In contrast, Ang II was associated with endothelial dysfunction in mice infused with 200, 400, and 1000 ng/kg/min Ang II that was dependent on both the dose and length of time of Ang II infusion (Figure 4). For example, 200 ng/kg/min Ang II was associated with a small rightward shift in the concentration-response curve to acetylcholine. However, 200 ng/kg/min Ang II had no effect (*P* > 0.05) on the relaxation produced by the highest concentration of acetylcholine (e.g., 100 mmol/L acetylcholine produced 89 ± 2 and 81 ± 4% relaxation on Day 14 and 28, respectively, in carotid arteries from mice infused with 200 ng/kg/min).

Similarly, relaxation to acetylcholine in carotid arteries from mice infused with 400 ng/kg/min Ang II was associated with a moderate degree of impairment of endothelial function on Day 14, however with time the degree of impairment was significantly greater (*P* < 0.05) on Day 28 (Figure 4). For example, 100 μmol/L acetylcholine produced 80 ± 6 and 55 ± 6% relaxation in carotid artery from mice infused with 400 ng/kg/min Ang II for 14 and 28 days, respectively.

Finally, 1000 ng/kg/min Ang II was associated with marked impairment of endothelial function in response to acetylcholine by Day 14 and was similar to that with infusion of 1000 ng/kg/min Ang II for 28 Days (Figure 4). For example, 1000 ng/kg/min Ang II was associated with 56 ± 7 and 58 ± 6% relaxation on Day 14 and 28, respectively. Endothelium-independent responses to nitroprusside were normal in control mice and mice infused with 50, 100, and 200 ng/kg/min, however responses to nitroprusside in carotid arteries from mice infused with 400 and 1000 ng/kg/min were significantly reduced as compared to those in control mice (data not shown).

### Ang II infusion was associated with vascular hypertrophy

**Figure 5:**
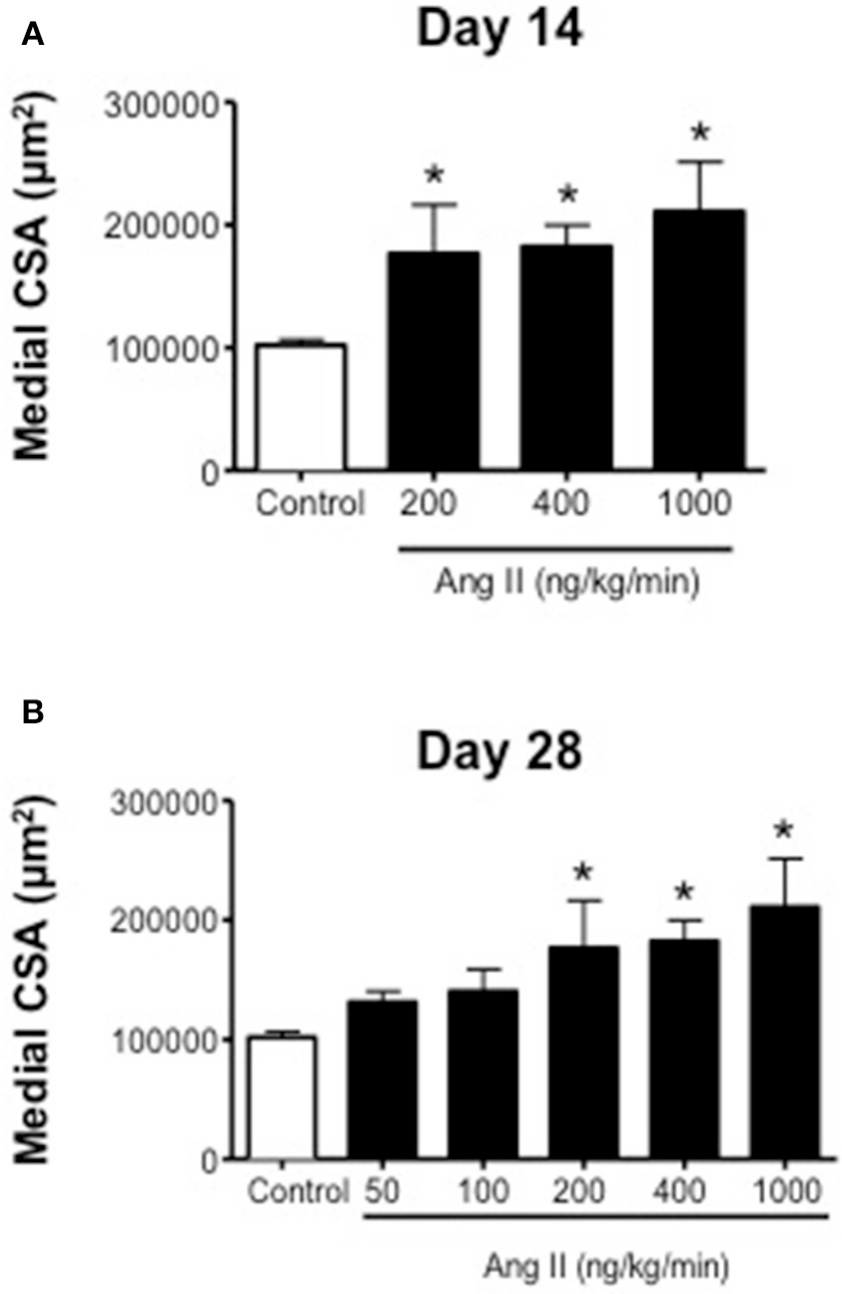
**Medial cross-sectional area (aortic) on (A) Day 14 and (B) Day 28 in control and Ang II infused mice**. Means ± SE; *n* = 3–6/group; ^*^P < 0.05 vs. Control.

Ang II was associated with vascular hypertrophy only with infusion of 200, 400, and 1000 ng/kg/min Ang II (Figure 5). Surprisingly, the effect of Ang II was for the most part all-or-none, with low doses of Ang II (i.e., 50 and 100 ng/kg/min) having no effect on medial CSA, whereas 200, 400, and 1000 ng/kg/min were all associated with significant increases in medial CSA. The degree of hypertrophy produced by all three doses was of similar magnitude and appeared to occur by Day 14 and did not appear to increase any further at Day 28 (Figure 5).

### Ang II infusion was associated with increases in plasma IL-6 levels and vascular macrophage content

**Figure 6:**
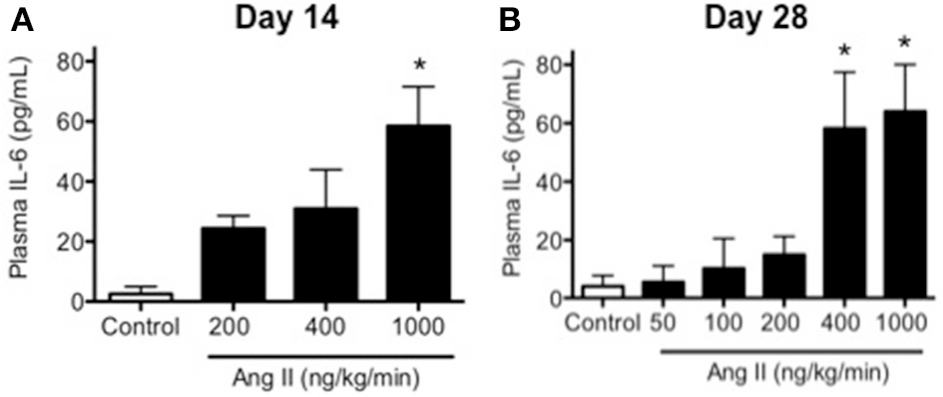
**Ang II infusion is associated with dose- and time-dependent increases in plasma IL-6**. Plasma IL-6 levels in control and Ang II infused mice on **(A)** Day 14 and **(B)** Day 28 as determined by ELISA. Means ± SE; *n* = 3–6/group; ^*^p < 0.05 vs. Control.

Plasma IL-6 levels were relatively low in control mice as would be predicted in the absence of inflammation and averaged 4 ± 3 pg/ml (Figure 6). Infusion of 200, 400, and 1000 ng/kg/min Ang II for 14 Days was associated with 6-, 8-, and 16-fold higher levels of IL-6 than that in control mice, e.g., IL-6 levels in mice infused with 200, 400, and 1000 ng/kg/min Ang II averaged 22 ± 4, 29 ± 8, and 64 ± 11 pg/ml, respectively (Figure 6).

**Figure 7:**
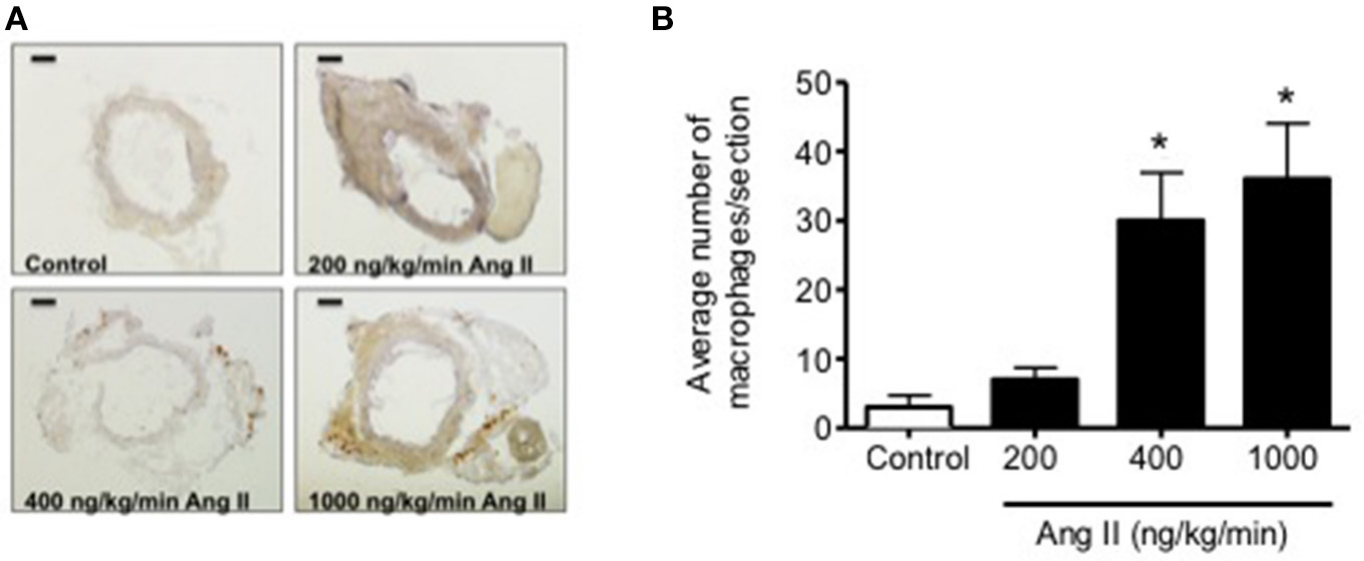
**(A)** Representative micrographs of vascular macrophage content in carotid arteries from control and Ang II infused (200, 400, and 1000 ng/kg/min) mice. **(B)** Quantification of vascular macrophage accumulation in carotid arteries from Control and Ang II-infused mice. Magnification: 40x; Scale bar = 500 μm. *n* = 3/group; ^*^p < 0.05 vs. control.

**Figure 8:**
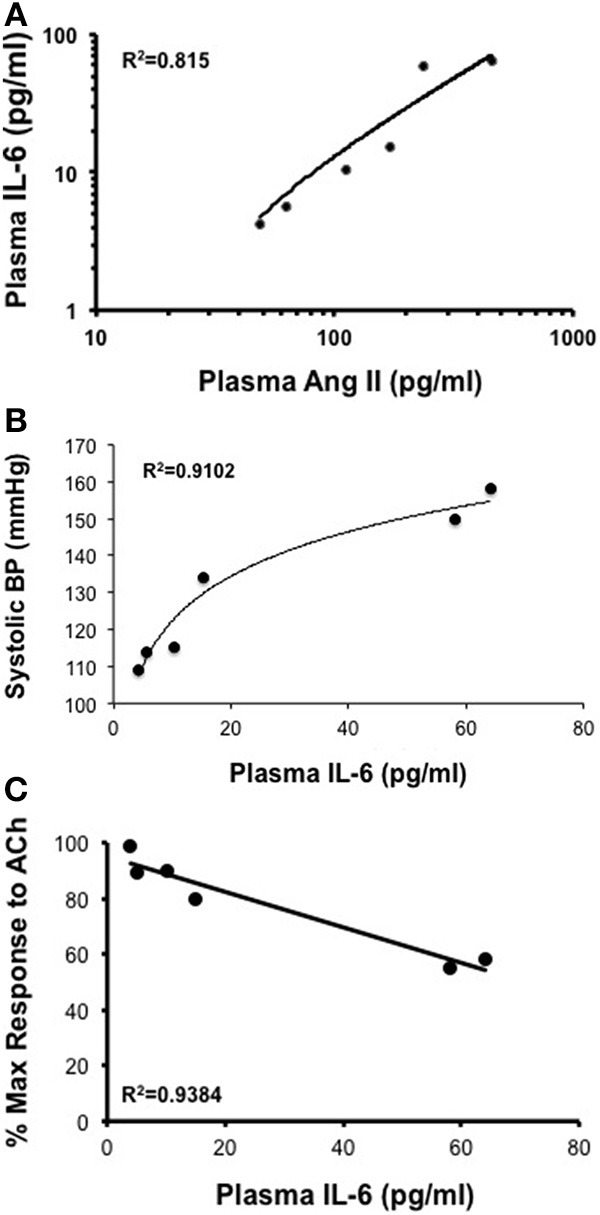
**(A,B)** While there is a direct correlation between plasma Ang II levels and plasma IL-6 and between plasma IL-6 levels and systolic blood pressure there was **(C)** an inverse relationship between plasma IL-6 and endothelial responses to acetylcholine (Ach) following Ang II infusion.

Infusion of 50, 100, 200, 400, and 1000 ng/kg/min Ang II for 28 Days was associated with plasma IL-6 levels that averaged 6 ± 6, 10 ± 10, 15 ± 6, 58 ± 19, and 64 ± 18 pg/ml, respectively. While plasma IL-6 levels in mice infused 50, 100, and 200 ng/kg/min were not different (*P* > 0.05) from that in control mice, infusion of 400 and 1000 ng/kg/min were significantly greater than that in control mice. More importantly, plasma IL-6 levels with infusion of 400 ng/kg/min Ang II for 28 Days was significantly greater (*P* < 0.05) than that with 14-Day infusion. Plasma IL-6 levels in mice infused with 1000 ng/kg/min Ang II for 28 Days were not different (*P* > 0.05) than that with infusion 1000 ng/kg/min Ang II for 14 Days (Figure 6). Interestingly, the number of vascular macrophages was significantly higher in mice infused with 400 and 1000 ng/kg/min Ang II as compared to that in control mice and mice infused with 200 ng/kg/min (Figure 7). Finally, when we examined the relationship between plasma IL-6 levels and plasma Ang II and systolic blood pressure we found that there was a strong correlation between the two (Figure 8). In contrast, there was a strong negative relationship between plasma IL-6 levels and the degree of endothelial function (Figure 8).

## Discussion

There are several important findings of the present study. First, Ang II over a wide range of doses in the mouse was associated with increases in systolic blood pressure that displayed both dose and time dependency. Second, the effective steady-state plasma concentration of Ang II that was required to produce a pressor response was ≥170 pg/ml, which was achievable with infusion of doses of Ang II greater than 200 ng/kg/min. Third, Ang II infusion was associated with minimal to no impairment of endothelial function at low doses (50–200 ng/kg/min) and marked impairment (nearly 50%) at the highest dose of Ang II (1000 ng/kg/min). Most interestingly, infusion of Ang II at 400 ng/kg/min was the only dose of Ang II that was associated with a temporal impairment of endothelial function. Fourth, plasma IL-6 levels and perivascular macrophage accumulation increased in parallel with blood pressure and plasma Ang II levels. More importantly, impairment of endothelial function was found to correlate directly with IL-6 levels and macrophage accumulation.

### Dose and temporal effects of Ang II infusion on blood pressure and plasma Ang II levels

While there is a fair amount of information regarding the effects of infusion of high doses of Ang II (e.g., 400–3600 ng/kg/min) (Kawada et al., 2002; Didion et al., 2005, 2009; Gavazzi et al., 2006; Lee et al., 2006; Guzik et al., 2007; Barhoumi et al., 2011), there is little to no information regarding the effects of lower doses (i.e., <400 ng/kg/min) on arterial pressure in the mouse. In the present study, we found that infusion of either 50 or 100 ng/kg/min Ang II was not associated with alterations in systolic blood pressure up to and including Day 28. The lack of effect of 50 and 100 ng/kg/min Ang II on blood pressure could be explained by fact that these doses were not associated with increases in plasma Ang II levels.

In contrast to the effects of lower doses, we found that infusion of intermediate doses of Ang II were associated with biphasic blood pressure responses. For example, 200 ng/kg/min had no effect on blood pressure up to Day 13, however blood pressure began to increase on Day 14 and by Days 21 and 28 blood pressure was significantly higher than baseline levels by at least 20 mmHg. Similarly, infusion of 400 ng/kg/min Ang II also produced a biphasic response, however the pressor response occurred earlier (by at least Day 7) and was of greater magnitude (~10 mmHg greater than that produced by 200 ng/kg/min). Our data with 200 and 400 ng/kg/min Ang II are consistent with previous findings using similar doses for shorter periods of time (<14 Days) (Kawada et al., 2002; Didion et al., 2009). Our findings also provide new information regarding long-term effects of Ang II infusion up to and including 28 Days.

Plasma Ang II levels were significantly higher in mice infused with 200 and 400 ng/kg/min Ang II compared to control mice, suggesting that these doses were sufficient to increase plasma Ang II concentrations and activate AT1 receptors. Our findings also suggest that there is a threshold of plasma Ang II of at least 170 pg/ml Ang II in order for Ang II to have any significant effect on blood pressure. While plasma Ang II levels tended to be higher in mice infused with 400 ng/kg/min they were not significantly different than those in mice infused with 200 ng/kg/min Ang II. It is difficult to know how such small incremental differences in plasma Ang II could have such differential effects on blood pressure, however one possibility may involve compartmentalized formation of endogenous Ang II in response to exogenous Ang II infusion. Indeed, Ang II infusion has been shown to stimulate de novo generation of intrarenal Ang II, which has been suggested to contribute to slow pressor response produced by low-dose Ang II infusion and may in part account for the differences in blood pressure produced by 200 and 400 ng/kg/min Ang II in the present study (Navar et al., 2011).

Infusion of 1000 ng/kg/min Ang II was associated with an immediate (by Day 3) increase in systolic blood pressure of nearly 40 mmHg, which was maintained throughout the 28-day infusion period. This is consistent with previous studies, including our own, in which 1000 ng/kg/min is typically associated with increases in arterial pressure of 40–50 mmHg (Didion et al., 2005; Schrader et al., 2007). In addition, the increase in arterial pressure achieved with 1000 ng/kg/min Ang II appears to represent an upper limit to which blood pressure can be increased with Ang II as infusion of higher doses (e.g., 3600 ng/kg/min) have not been found to increase blood pressure to any greater extent than that achieved with 1000 ng/kg/min (Lee et al., 2006).

As one might predict, infusion of 1000 ng/kg/min Ang II was associated with the largest increase in plasma Ang II levels (nearly 450 pg/ml). While we did not measure plasma Ang II levels other than on Day 28 we would suggest that the levels of Ang II measured on Day 28 most likely represents plasma steady-state levels that are achieved with long-term infusion of Ang II. Surprisingly, the majority of studies using the Ang II infusion model in mice do not measure and/or report plasma Ang II levels (Bush et al., 2000; Crowley et al., 2005, 2006; Didion et al., 2005; Lee et al., 2006; Guzik et al., 2007; Madhur et al., 2010; Barhoumi et al., 2011). In the present study, we measured plasma Ang II levels in all our experimental groups and found that there was a direct correlation between plasma Ang II levels and arterial pressure consistent with observations in other species, including man (Chinn and Dusterdieck, 1972; Bean et al., 1979; Brown et al., 1981; Cholewa and Mattson, 2001; Beevers et al., 2008). The present study is the first to our knowledge to characterize the plasma Ang II concentration-blood pressure response curve following subcutaneous infusion of Ang II in the mouse.

Previous studies have shown that the increase in blood pressure produced by Ang II is mediated in large part by increases in oxidative stress, as scavengers of oxidative stress such as Tempol limit the increase in pressure produced by Ang II infusion (Kawada et al., 2002). The initial increase in blood pressure in response to high pressor doses of Ang II appears to be mediated by Nox2-derived superoxide (Wang et al., 2006). In contrast, Nox1-derived superoxide appears to mediate the long-term effects of Ang II on blood pressure as Nox1 deficiency is associated with a rapid increase in blood pressure followed by a return toward baseline levels (Matsuno et al., 2005; Gavazzi et al., 2006). Although we did not examine the role of oxidative stress in the development of hypertension in present study, we would speculate that both Nox1- and Nox2-derived superoxide contribute to independent phases of the hypertension produced in response to Ang II and are mediated via combined and/or temporal activation of vascular, central, as well as renal AT1 receptors (Zimmerman et al., 2002; Ryan et al., 2004; Crowley et al., 2005, 2006).

### Dose and temporal effects of Ang II on plasma IL-6 levels and vascular function and macrophage accumulation

In addition to hypertension, Ang II can produce endothelial dysfunction in a number of blood vessels (e.g., aorta, carotid artery, cerebral arterioles) in several species, including mice (Rajagoplan et al., 1996; Su et al., 1998; Didion et al., 2000, 2002, 2005; Wang et al., 2001; Didion and Faraci, 2003; Faraci et al., 2006; Guzik et al., 2007; Barhoumi et al., 2011). Both pharmacological and genetic approaches have demonstrated that NO (derived primarily from eNOS) is a major mediator of endothelium-dependent relaxation in mouse carotid artery (Faraci et al., 1998). Oxidative stress, such as increases in vascular superoxide levels, limits NO bioavailability and endothelium-dependent relaxation (Didion et al., 2005). We, and others, have shown that interventions directed at reducing vascular superoxide levels produced in response to a number of stimuli, including Ang II, are very effective in restoring NO and improving endothelial function (Didion and Faraci, 2002, 2005; Didion et al., 2005). Additionally, we have shown that the endothelial dysfunction produced in response to Ang II is mediated primarily through AT1 receptors (Ryan et al., 2004).

High doses of Ang II (i.e., ≥ 490 ng/kg/min) produce marked impairment of endothelial function (Bean et al., 1979; Didion et al., 2005, 2009; Guzik et al., 2007; Schrader et al., 2007; Madhur et al., 2010; Barhoumi et al., 2011), however the effects of lower doses of Ang II on vascular function and structure are poorly defined. Thus, in order to elucidate the temporal processes involved in the development of vascular hypertrophy and endothelial dysfunction in response to Ang II, we examined the effect of multiple doses and time points of Ang II infusion on both of these processes. We elected to examine vascular function in carotid artery, as endothelial dysfunction in this blood vessel is major risk factor for ischemic stroke (Roger et al., 2012; Kernan et al., 2014).

In the present study, we found that, like blood pressure, the degree of endothelial dysfunction produced by Ang II was both dose- and time-dependent. For example, low doses of Ang II (e.g., 50 and 100 ng/kg/min Ang II), which did not effect on blood pressure, were also without effect on endothelial function and vascular structure. The lack of effect of these two doses is most likely reflective of the fact that they were not associated with increases in plasma Ang II levels and thus not sufficient to activate AT1 receptor signaling. Similarly, doses of Ang II that were associated with slowly developing increases in arterial pressure and plasma Ang II levels (such as 200 ng/kg/min Ang II) were surprisingly not associated with alterations in endothelial function despite a marked increase in vascular hypertrophy. Interestingly, infusion of 200 ng/kg/min Ang II was not associated with alterations in plasma IL-6 levels or vascular macrophage content compared with that in control mice, suggesting that the increase in blood pressure at these doses occurs independently of increases in inflammatory cells and cytokines. Additionally, our data with 200 ng/kg/min Ang II also suggest that vascular hypertrophy precedes the development of hypertension, consistent with the idea that the effect of non-pressor doses of Ang II on vascular structure are due to direct effects of Ang II and independent of blood pressure (Owens and Schwartz, 1982; Berk et al., 1989; Griffin et al., 1991; Su et al., 1998).

Perhaps of equal importance, we found that infusion of 400 ng/kg/min Ang II was the only dose in the present study that was associated with temporal impairment of endothelial function. Ang II at this dose was associated with minimal impairment of endothelial function as evidenced by the reduced response to acetylcholine at Day 14. However, endothelial function was impaired to a much greater extent (i.e., a reduction of approximately 50% in the response to acetylcholine) on Day 28 in mice that were infused 400 ng/kg/min Ang II. The degree of impairment associated with 28-day infusion of 400 ng/kg/min Ang II was very similar to the degree of endothelial dysfunction produced with either 14 or 28 day infusion of 1000 ng/kg/min Ang II, both of which were associated with increases in IL-6 and vascular macrophage levels. Taken together, these findings suggest that inflammation is a major contributor to endothelial dysfunction produced in response to Ang II and is most likely reflective of the degree of hypertension produced by Ang II and/or the length of time hypertension is present.

Consistent with previous studies, infusion of 1000 ng/kg/min of Ang II produced approximately 50% impairment of endothelium-dependent relaxation in response to acetylcholine as early as Day 14 (Didion et al., 2005; Schrader et al., 2007). The degree of impairment produced by 1000 ng/kg/min was not affected by the length of time in which Ang II was infused as it was found to be similar on Day 28 as that observed on Day 14. Ang II is known to increase levels of proinflammatory cytokines, such as IL-6, IL-17, and IL-18, in vascular cells (Faraci et al., 1998; Funakoshi et al., 1999; Han et al., 1999; Liu et al., 2003; Sahar et al., 2005; Schrader et al., 2007; Johnson et al., 2013). Consistent with this concept, we found in the present study that IL-6 levels were nearly 60-fold higher in mice infused with 1000 ng/kg/min Ang II than that in control mice. Moreover, the increase in plasma IL-6 correlated directly with the degree of endothelial dysfunction and vascular macrophage content.

Functional effects of Ang II-induced increases in IL-6 includes downstream activation of STAT3 (Schieffer et al., 2000; Johnson et al., 2013). STAT3 activation has been shown to have a number of effects, including inhibition of eNOS promoter activity and reductions in eNOS gene expression (Saura et al., 2006), thereby providing a mechanistic link by which IL-6 serves to produce endothelial dysfunction. IL-6 has also been shown to increase expression of AT1 receptors in vascular muscle, which would serve to promote Ang II signaling including increased NFκB activation and IL-6 gene transcription (Han et al., 1999), which would then further enhance IL-6 mediated-reductions in eNOS expression and promote increases in oxidative stress (Wassmann et al., 2004). While we did not examine the contribution of oxidative stress to the impairment of endothelial function, previous studies, including our own, have shown that increases in oxidative stress, particularly Nox2-derived superoxide, contribute to endothelial dysfunction produced by Ang II (Rajagoplan et al., 1996; Didion et al., 2005, 2009; Schrader et al., 2007). While it is difficult to differentiate whether vascular or infiltrating immune cells (as evidence by the increase in vascular macrophage content) associated with 400 and 1000 ng/kg/min Ang II) contributed to the increase in plasma IL-6 which then serves to promote increases in oxidative stress, we have shown previously that vascular IL-6 expression contributes to Ang II-induced impairment of endothelial responses (Schrader et al., 2007).

In conclusion, the findings from our study provide a comprehensive examination of the effects of Ang II on both blood pressure and endothelial function in the mouse. Our findings demonstrate that Ang II produces hypertension and endothelial dysfunction in a dose- and time-dependent manner. These findings are important as they serve to delienate the temporal events associated with the development of Ang II-induced endothelial dysfunction. Our data also demonstrate along with increases in arterial pressure that there is a strong correlation between increases in IL-6 and vascular macrophage accumulation and the degree of endothelial dysfunction produced by Ang II. It will be important to determine whether inflammation promotes oxidative stress or vice versa and whether targeting one or both pathways will be most effective in limiting the negative effects of Ang II on vascular function in human hypertension. This is especially important considering that plasma IL-6 correlates with negative cardiovascular outcomes in humans (Ikeda et al., 1992; Ridker et al., 2000a,b).

### Conflict of interest statement

The authors declare that the research was conducted in the absence of any commercial or financial relationships that could be construed as a potential conflict of interest.
